# Comprehensive risk factor predictions for 3-year survival among HIV-associated and disseminated cryptococcosis involving lungs and central nervous system

**DOI:** 10.1007/s15010-024-02237-6

**Published:** 2024-04-13

**Authors:** Luling Wu, Xuemin Fu, Benno Pütz, Renfang Zhang, Li Liu, Wei Song, Ling Weng, Yueming Shao, Zhihang Zheng, Jingna Xun, Ximei Han, Ting Wang, Yinzhong Shen, Hongzhou Lu, Bertram Müller-Myhsok, Jun Chen

**Affiliations:** 1 Institute of Antibiotics, Huashan Hospital, Fudan University, Shanghai, China; 2 Department of Infectious Diseases and Immunology, Shanghai Public Health Clinical Center, Fudan University, Shanghai, China; 3 Research Group Statistical Genetics, Max Planck Institute of Psychiatry, Munich, Germany; 4 Department of Respiratory Medicine, Fuzhou Pulmonary Hospital, Fuzhou, Fujian China; 5 Department of Infectious Diseases and Nursing Research Institution, National Clinical Research Center for Infectious Diseases, The Third People’s Hospital of Shenzhen, Shenzhen, China

**Keywords:** HIV/AIDS, Disseminated cryptococcosis, Three-year survival-related predictions, Antiretroviral and antifungal drug therapies

## Abstract

**Background:**

The global mortality rate resulting from HIV-associated cryptococcal disease is remarkably elevated, particularly in severe cases with dissemination to the lungs and central nervous system (CNS). Regrettably, there is a dearth of predictive analysis regarding long-term survival, and few studies have conducted longitudinal follow-up assessments for comparing anti-HIV and antifungal treatments.

**Methods:**

A cohort of 83 patients with HIV-related disseminated cryptococcosis involving the lung and CNS was studied for 3 years to examine survival. Comparative analysis of clinical and immunological parameters was performed between deceased and surviving individuals. Subsequently, multivariate Cox regression models were utilized to validate mortality predictions at 12, 24, and 36 months.

**Results:**

Observed plasma cytokine levels before treatment were significantly lower for IL-1RA (*p* < 0.001) and MCP-1 (*p* < 0.05) when in the survivor group. Incorporating plasma levels of IL-1RA, IL-6, and high-risk CURB-65 score demonstrated the highest area under curve (AUC) value (0.96) for predicting 1-year mortality. For 1-, 2- and 3-year predictions, the single-factor model with IL-1RA demonstrated superior performance compared to all multiple-variate models (AUC = 0.95/0.78/0.78).

**Conclusions:**

IL-1RA is a biomarker for predicting 3-year survival. Further investigations to explore the pathogenetic role of IL-1RA in HIV-associated disseminated cryptococcosis and as a potential therapeutic target are warranted.

**Supplementary Information:**

The online version contains supplementary material available at 10.1007/s15010-024-02237-6.

## Introduction

Cryptococcosisca is a global opportunistic infection caused by *Cryptococcus neoformans* and *Cryptococcus gattii* yeasts [1]. The symptoms of cryptococcosis vary widely, from mild pulmonary cases with fever, chills, and cough, to life-threatening disseminated infections, especially spreading over the central nervous system (CNS), causing headache, cranial neuropathies, altered mentation, memory loss, and cryptococcal meningitis (CM) [2]. Despite the widespread rollout of antiretroviral therapy (ART), mortality of people living with HIV (PLWH) with CI, which has been the second-leading cause of human immunodeficiency virus (HIV)-related deaths after tuberculosis, remains high [3, 4]. Globally CM results in approximately 112,000 deaths per year in AIDS adults [4]. Apart from fatality, CM survivors may suffer persistent poor prognoses, such as neurological impairments, disability, and poor life quality [5]. Previous studies found that 1-year mortality of PLWH with CM in Malawi who were only treated with fluconazole even exceeded 78% [6]. As described, disseminated cryptococcosis involving the lungs and CNS exhibited poorer outcomes and inadequate treatment response compared to CM alone [7]. Altered neurological status, elevated fungal burden in cerebrospinal fluid (CSF), reduced CD4^+^ T cell counts, and advanced age all were essential risk factors related to short-term (within 1 year) mortality for CM patients with HIV infections. It is possible for patients surviving more than 6 months who suffered from CM with AIDS to survive for more than 5 years [3]. Studies focusing on the long-time risk factor assessment and predictions associated with death in PLWH with CM are highly limited. Up to now, except for a few short-term studies [6], it is also a challenging problem to collect long-term longitudinal post-treated data on large cohorts with cryptococcal and HIV-related patients.

To address this gap and better characterize the long-term mortality-related risk factors and prognosis, we collected a clinical cohort including survivors and non-survivors over 3 years of PLWH with disseminated cryptococcosis involving the lung and CNS. In addition, we collected clinical as well as immunological features, constructed multivariate predictive models related to 3-year survival, and assessed the post-treated outcomes with different ARTs and antifungal treatments at several follow-up time points.

## Methods

### Ethics statement

The study was approved by the Research Ethics Committee of the Shanghai Public Health Clinical Center (approval number 2020-Y112-01) and complied with all relevant ethical regulations.

### Cohort design and sample collection

**Fig. 1:**
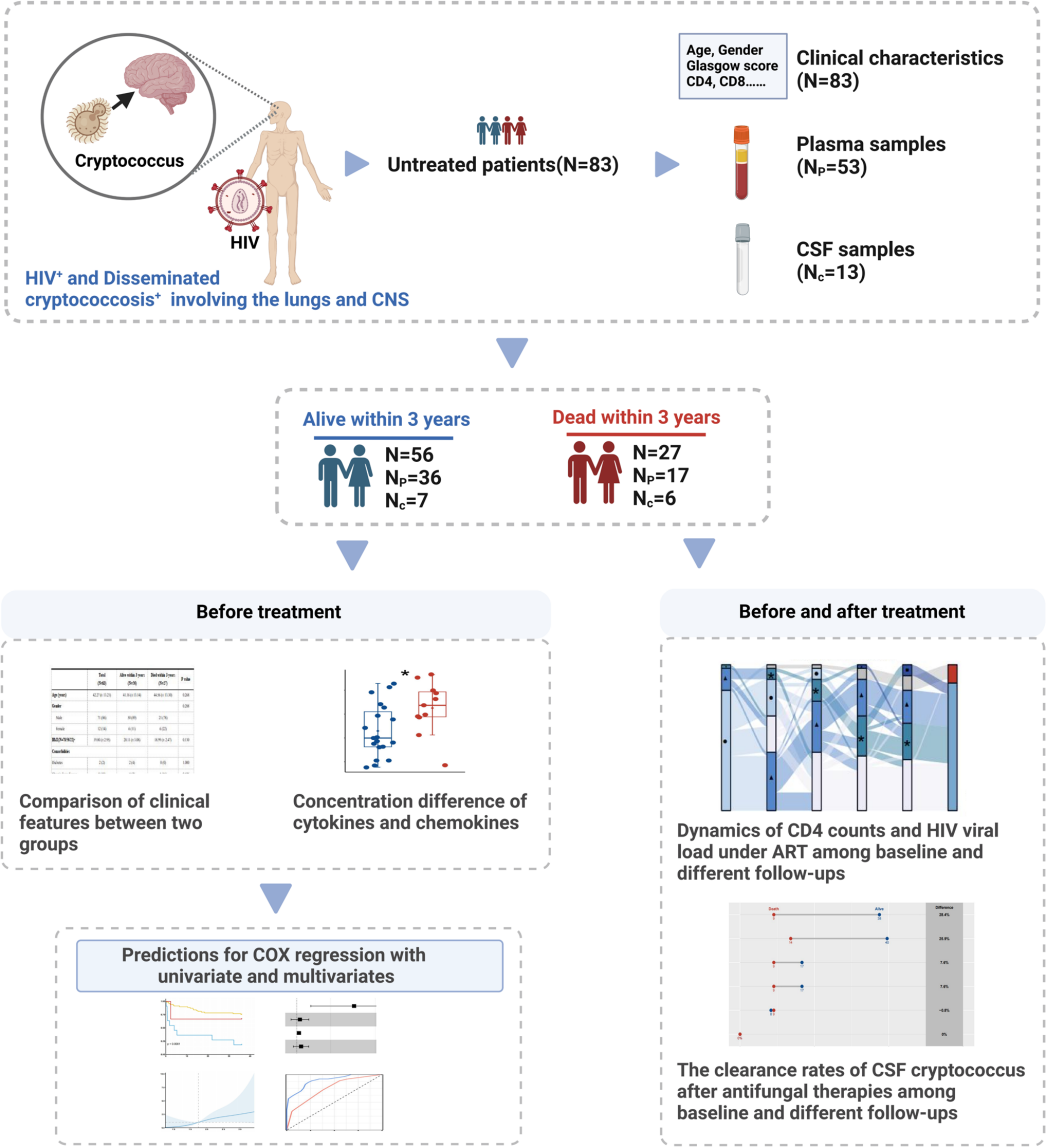
Flowchart of comprehensive analysis for risk factor predictions related to mortality within 3 years and longitudinal dynamics under treatments among HIV-associated and disseminated cryptococcosis involving the lungs and CNS. N_P_ represents the number of plasma samples, and N_C_ means the number of CSF samples. Figure 1 was created with biorender.com. Abbreviations: CNS, Central nervous system; CSF, Cerebrospinal fluid; ART, Antiretroviral therapy

Disseminated cryptococcosis is characterized by the identification of Cryptococcus in (1) positive blood cultures or (2) positive cultures or positive cryptococcal antigen (CrAg) titers from a minimum of two separate body sites [8]. In this study, a cohort including 83 untreated patients with HIV-associated disseminated cryptococcosis involving the lungs and CNS were enrolled at the Department of Infection and Immunology, Shanghai Public Health Clinical Center, from January 31, 2015, to December 31, 2022. Apart from demographic data and clinical characteristics of all patients, routine laboratory test results, imaging features, and the 3-year survival status were all involved and followed (Fig. 1). In the cohort, the data were excluded if missingness was more than 20%. Within 24 h of admission, all patients underwent assessments using the Glasgow Coma Scale (GCS) [9], Sequential Organ Failure Assessment (SOFA) score [10], and confusion, urea nitrogen, respiratory rate, blood pressure, and age ≥ 65 years (CURB-65) severity score [10] to evaluate the severity of their disease. As described in Supplementary Method, all antifungal therapies were initiated as soon as the participants were diagnosed with cryptococcosis. ARTs were started after either the cryptococcal infection in the CSF was cleared totally or at least 4–6 weeks with antifungal treatment [11]. Following established workflows (Supplementary Fig. 1), longitudinal data on ARTs were collected, encompassing CD4^+^T cell counts and plasma HIV viral loads over 3 years, with follow-up at various time points (0 [baseline], 6, 12, 24, and 36 months). Simultaneously, 1-year clearance rates (negative/total) of CSF Cryptococcus were documented at different follow-up time points (0 [baseline], 0.5, 1, 3, 6, and 12 months).

Before all treatments, plasma and CSF samples were transported to the specimen bank of Infection and Immunology, Shanghai Public Health Clinical Center. After both centrifugations at 1000 g for 20 min and divisions into aliquots, samples were stored at –80℃ until they were ready for further experimentation. All samples were obtained from the specimen bank, and qualified samples were screened for subsequent testing.

### Plasma and CSF testing and analysis

A total of 53 (alive/dead, 36/17) plasma samples and 13 (7/6) CSF samples were selected and measured using the multiplex ELISA method (Bio-Plex Pro Human Cytokine 27-plex Assay, catalog no: #M500KCAF0Y, Bio-Rad Laboratories, Inc., Hercules, CA, USA). The following molecules were determined: interleukins (IL)-1β, IL-2, IL-4, IL-5, IL-6, IL-7, IL-8, IL-9, IL-10, IL-12p70, IL-13, IL-15, IL-17, IFNγ, tumor necrosis factor-α (TNFα), interferon-inducible protein-10 (IP-10), IL-1RA, monocyte chemoattractant protein-1 (MCP-1), macrophage inflammatory protein-1α (MIP-1α), macrophage inflammatory protein-1 β (MIP-1β), platelet-derived growth factor-BB (PDGF-BB), RANTES, granulocyte–macrophage colony-stimulating factor (GM-CSF), granulocyte colony-stimulating factor (G-CSF), vasoactive endothelial growth factor (VEGF), fibroblast growth factor (FGF), and eotaxin. To quantify cytokine and chemokine levels, the Bio-Plex Manager software was employed to analyze the assay data. Cytokine and chemokine values below the experimentally determined detection threshold were recorded as zero, signifying undetectable levels. Conversely, quantities surpassing the upper limit of quantification of the standard curve were deemed outside the assay range and assigned the maximal value on the curve [12].

### Statistical analysis

Categorical variables were expressed as frequencies and percentages, whereas continuous variables were presented as mean [standard deviation (SD)] or median [interquartile range (IQR)]. For normally distributed numerical data, comparisons between groups were implemented using a paired or unpaired *t*-test, otherwise utilizing the Mann–Whitney *U* test. Two-sided Fisher’s exact test (or *χ*^2^-test) was used to compare categorical variables (proportions between groups). A difference with *p* < 0.05 was considered to be statistically significant.

All cytokine values which are 1.5 IQR above Q3 or below Q1 were identified as outliers and subsequently imputed using random forest (RF) models. Then, all cytokines data were log_10_ transformed, and to avoid issues related to log-transforming zero, all values below the detection limit were imputed by half the detection limit [13].

### Survival analyses with single variables

Variables with a significance level of less than 0.1 in univariate analysis were subjected to further analysis. Kaplan–Meier survival estimates were calculated for categorizable variables among untreated HIV patients censored at survival time within 36 months. To evaluate the impact of cytokine level on 36-month survival, a univariate Cox model with restricted cubic splines (RCS) was constructed. The 95% CI was derived through bootstrap resampling. For all survival analyses, differences for specific subsets of data were compared using the log-rank test. A *p*-value < 0.05 was regarded as statistically significant.

### Multivariate prediction models with Cox regression analysis

All data were partitioned into the training and the test subsets at a 70:30 ratio. Cox regression models were constructed using the training subset, and the formulated models were verified on the test division to quantify the test performance. Variables with significance (*p* < 0.05) in univariate survival analysis on training data underwent further multivariate Cox regressions. Subsequently, a total of four models were established stepwise based on increasing false discovery rate (FDR) values among selected variables. Multivariate analysis incorporating multiphase hazard analysis was used to identify survival predictors. We assessed the discrimination and calibration ability of the models based on the area under the receiver operating characteristic curve (AUC) at months 12, 24, and 36 for survival.

All analyses were performed utilizing R version 4.2.1 [14] and the following packages: tidyverse [15], ggplot2 [16], missForest [17], survival [18], survminer [19], rms [20], and survival receiver operating characteristic (ROC) curve [21]. All flowchart plots were drawn using biorender.

## Results

### Clinical characteristics of study participants

To investigate the association between clinical and immunological risk factors and the prolonged survival of disseminated cryptococcosis and HIV-related disease, we initiated a study with the largest cohort to date. The study cohort comprised 83 adult patients with HIV who exhibited cryptococcosis impacting both the lungs and the CNS. Thorough data encompassing demographics and clinical characteristics were diligently collected at the time of diagnosis, prior to the initiation of any treatment interventions (Fig. 1). Among the cohort, males constituted the majority (85.54%), and the mean age was 42 years. The observed 3-year mortality rate was 32.53% (27/83) (Table 1). Comparisons between the deceased and the survivors within the 3-year period revealed no significant differences in terms of gender, age, and body mass index (BMI). Patients without comorbidities, such as malignancy, diabetes, and chronic diseases, were 84.34% (70 of 83 patients) and remained consistent across both groups. Common symptoms reported included fever (69.88%), headache (45.78%), and vomiting (32.53%), as documented in Supplementary Table 1. There were notable similarities in most of the clinical variables between the survivor and non-survivor groups. Intriguingly, within our cohort, the baseline median CD4^+^ T cell counts were identical for all patients (21 cells/*μ*L). However, significant between-group differences were observed for variables such as intracranial pressure (ICP), GCS, and CURB-65 severity score. Notably, compared with extremely high ICP (> 300 mmH_2_O), patients with ICP values ranging from 181 to 330 mmH_2_O prior to treatment had the highest proportion of mortality within 3 years (66.67%). Similar trends were observed for GCS and CURB-65 scores, indicating that higher severity scores at 24-h admission were associated with increased 3-year mortality rates (Table 1).

**Table 1:** Demographic and clinical characteristics of untreated HIV-associated and disseminated cryptococcosis patients involving the lungs and CNS between alive and dead within 3 years

	Total (*N* = 83)	Alive within 3 years (*N* = 56)	Dead within 3 years (*N* = 27)	*P* values
Age, years, mean (SD)	42.27 (13.21)	41.16 (13.14)	44.56 (13.30)	0.27
Gender				0.29
Male	71 (85.54)	50 (89.29)	21 (77.78)	
Female	12 (14.46)	6 (10.71)	6 (22.22)	
BMI, kg/m^2^, mean (SD) (*N* = 78/56/22)^a^	19.80 (2.95)	20.11 (3.08)	18.99 (2.47)	0.13
Comorbidities				0.41
Diabetes	2 (2.41)	2 (3.57)	0 (0)	
Chronic liver disease	8 (9.64)	4 (7.14)	4 (14.81)	
Chronic kidney disease	1 (1.20)	0 (0)	1 (3.70)	
Malignancy	3 (3.61)	1 (1.79)	2 (7.41)	
None of the above	70 (84.34)	49 (87.50)	21 (77.78)	
Respiratory symptoms	23 (27.71)	12 (21.43)	11 (40.74)	0.07
Neurological symptoms	63 (75.90)	43 (76.79)	20 (74.07)	0.79
HIV-associated opportunistic infections				0.27
Tuberculosis	12 (14.46)	9 (16.07)	3 (11.11)	
CMV	4 (4.82)	4 (7.14)	0 (0)	
PCP	10 (12.05)	4 (7.14)	6 (22.22)	
NTM	9 (10.84)	7 (12.50)	2 (7.41)	
Syphilis	8 (9.64)	5 (8.93)	3 (11.11)	
Others	5 (6.02)	2 (3.57)	3 (11.11)	
CD4, cells/μL, median (IQR) (*N* = 81/54/27)^a^	21 (7.50–35)	21 (6–45.25)	21 (10–32)	0.98
CD8, cells/μL, mean (SD) (*N* = 79/52/27)^a^	376.19 (306.62)	403.85 (340.88)	322.93 (222.79)	0.53
WBC, 10^^^9/L, mean (SD)	5.55 (4.15)	5.23 (2.82)	6.20 (6.07)	0.92
N, 10^^^9/L, median (IQR)	4.47 (2.09–5.72)	3.43 (2–5.95)	3.27 (2.61–5.71)	0.56
L, 10^^^9/L, median (IQR)	0.50 (0.32–0.73)	0.54 (0.33–0.75)	0.42 (0.29–0.64)	0.21
HB, g/L, mean (SD)	115.82 (19.82)	117.91 (18.37)	111.48 (22.27)	0.17
ALB, g/L, mean (SD)	36.35 (7.67)	36.33 (4.91)	36.39 (11.59)	0.46
Serum CrAg titers (*N* = 70/48/22)^a^				0.64
[80,320]	12 (14.46)	10 (17.86)	2 (7.41)	
[640,1280]	21 (25.30)	14 (25)	7 (25.93)	
2560	37 (44.58)	24 (42.86)	13 (48.15)	
ICP, mmH_2_O (*N* = 81/56/25)^a^				0.01^*^
ICP values < 80	5 (6.02)	5 (8.93)	0 (0)	0.32
ICP values between 80 and 180	16 (19.28)	14 (25)	2 (7.41)	0.07
ICP values between 181 and 330	39 (46.99)	21 (37.50)	18 (66.67)	0.01^*^
ICP values > 330	21 (25.30)	16 (28.57)	5 (18.52)	0.49
CSF total protein, g/L, mean (SD) (*N* = 82/56/26)^a^	559.56 (473.42)	562.39 (467.90)	553.47 (494.45)	0.75
CSF glucose, mmol/L, mean (SD) (*N* = 82/56/26)^a^	2.34 (0.95)	2.23 (0.96)	2.57 (0.91)	0.13
Glasgow Coma Scale				0.03^*^
Mild[13,15]	51 (61.45)	37 (66.07)	14 (51.85)	0.21
Moderate[9,12]	6 (7.23)	6 (10.71)	0 (0)	0.17
Severe[3,8]	26 (31.33)	13 (23.21)	13 (48.15)	0.02^*^
CURB-65 severity score				<0.001^***^
Low risk[0,1]	69 (83.13)	52 (92.86)	17 (62.96)	0.002^**^
Moderate risk[2]	3 (3.61)	2 (3.57)	1 (3.70)	1.00
High risk[3,5]	11 (13.25)	2 (3.57)	9 (33.33)	0.001^**^
SOFA score				0.35
SOFA < 2	40 (48.19)	29 (51.79)	11 (40.74)	
SOFA ≥ 2	43 (51.81)	27 (48.21)	16 (59.26)	

^a^Available number of patients: In total/for alive patients within 3 years/for the dead within 3 years

Categorical variables are presented as number (frequency %)

*P*-values are calculated by Mann–Whitney U test for continuous variables and Chi-square test (or Fisher’s exact test as appropriate) for categorical variables (*p < 0.05; **p < 0.01, ***p < 0.001)

All severity evaluation scores are calculated based on the maximum values of the patients from the admission period up to 24 h

Abbreviations: *ALB* albumin, *ART* antiretroviral therapy, *BMI* body mass index, *CMV* cytomegalovirus, *CNS* central nervous system, *CrAg* cryptococcal antigen, *CSF* cerebrospinal fluid, *CURB-65* confusion, urea nitrogen, respiratory rate, blood pressure, and age ≥ 65 years, *HB* hemoglobin, *ICP* intracranial pressure, *L* lymphocyte, *N* neutrophil, *NTM* non-tuberculosis mycobacteria, *PCP* pneumocystis carinii pneumonia, *SOFA* Sequential Organ Failure Assessment, *WBC* white blood cell

### Cytokine/chemokine-associated with 3-year survival

**Fig. 2:**
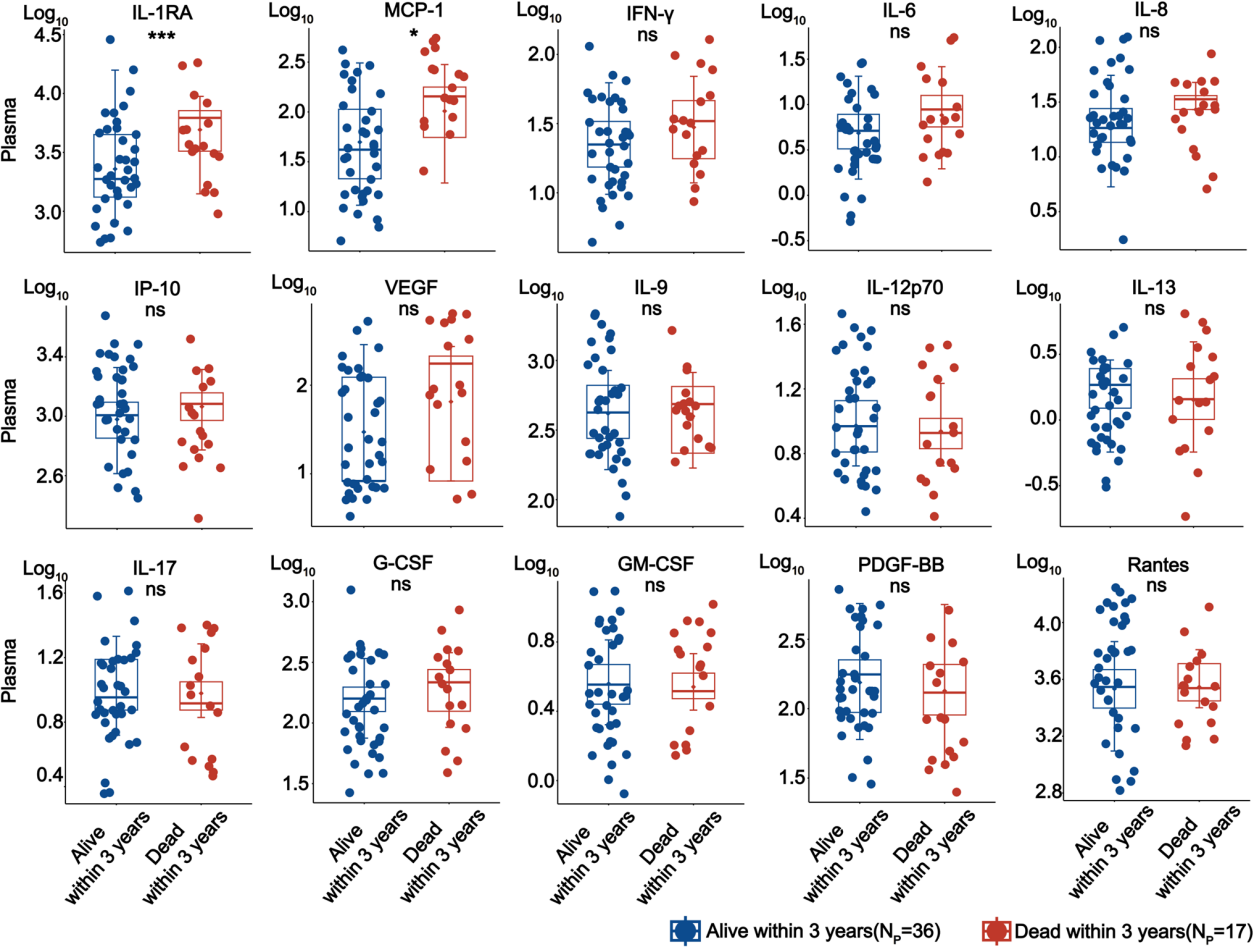
Comparisons of plasma cytokine and chemokine concentrations between the two groups of untreated patients who are alive or dead over 3 years. All cytokine and chemokine values are in picograms per milliliter (pg/ml) and log-transformed before comparisons. The *p* values are calculated with the *t*-test (*p < 0.05; **p < 0.01; ***p < 0.001; ns means not significant). Abbreviations: G-CSF, Granulocyte colony-stimulating factor; GM-CSF, Granulocyte–macrophage colony-stimulating factor; IL-1RA, Interleukin 1 receptor antagonist; MCP-1, Monocyte chemoattractant protein-1; PDGF-BB, Platelet-derived growth factor-BB; IP-10, Interferon-inducible protein-10; VEGF, Vasoactive endothelial growth factor

Cytokines play a crucial role in mediating antiviral and antifungal responses. To gain insight into the association between cytokines and 3-year survival, we collected plasma and CSF samples from untreated survivor and non-survivor groups. Using a multiplex ELISA assay, we measured 27 common cytokines and chemokines in both sample types. Significantly elevated levels of two plasma cytokines, IL-1RA (3.35 pg/ml vs 3.69 pg/ml, *p* < 0.001) and MCP-1 (1.69 pg/ml vs 2.01 pg/ml, *p* < 0.05), were observed in the non-survivor group compared to survivors (Fig. 2 and Supplementary Fig. 2). A similar trend was observed in CSF samples, although no statistical significance was found (Supplementary Fig. 3). In contrast, only one cytokine, IL-10 (*p* < 0.05), was detected in the CSF, with statistically higher levels observed in the non-survivor group compared to the survivors (Supplementary Fig. 3). No cytokines were consistently identified in both plasma and CSF samples.

### Survival analysis and predictions

**Fig. 3:**
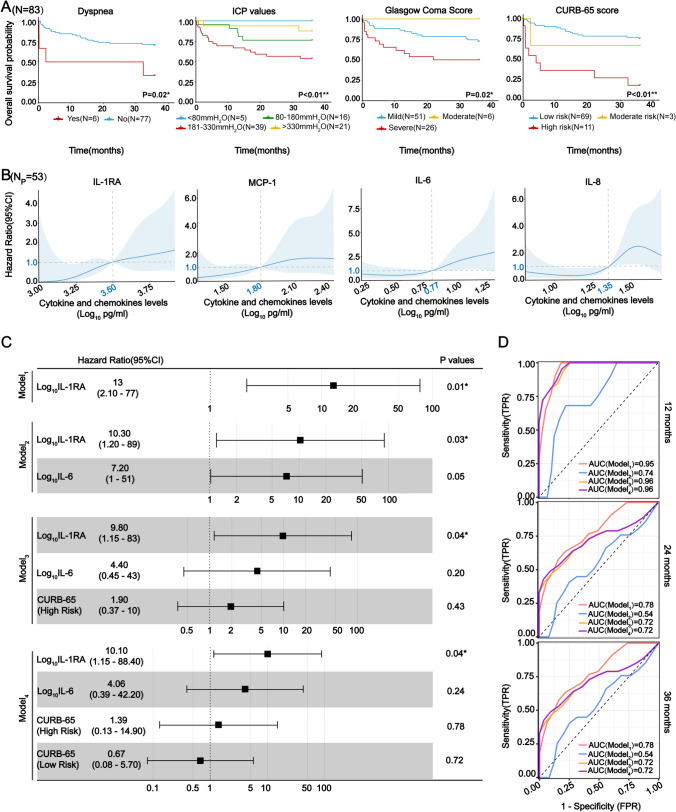
Survival analysis with univariate and multiple variants. **a** Survival analysis with the Kaplan–Meier (KM) estimator obtained from the clinical features significantly different in univariate analysis. All shown *p* values are calculated based on the log-rank test. N denotes the total number of patients. **b** Restricted cubic spline (RCS) curves selected from the cytokines and chemokines of plasma which are significantly different in univariate analysis. The numbers in blue represent the cutoff value at which the effect of the cytokine or chemokine levels on mortality changes. Solid lines indicate hazard ratio values, and blue-shaded areas show 95% confidence intervals. N_P_ represents the number of plasma samples. **c** Forest plots for different predictions involving multivariate Cox proportional hazards models (N_T1_ = 36). The data are split into a training set (N_T1_) and a validation set (N_T2_) using a 70:30 ratio. Model_1_ includes IL-RA as the sole predictor variable. In Model_2_, IL-6 adds to the list of predictors along with IL-RA. Model_3_ incorporates both IL-RA and IL-6 as well as a high-risk CURB-65 score. Model_4_ includes all the variables from Model 3 and adds a low-risk CURB-65 score as another predictor. All models are stepwise established on all involved variables according to FDR values (Supplement 6). The *p* values are evaluated with Wald test. **d** Receiver operating characteristic curve (ROC) plots of four multivariate models (N_T2_ = 17). The AUC values shown represent the prediction performances of different models for the mortalities at 12 months, 24 months, and 36 months separately (*p < 0.05; **p < 0.01). Abbreviations: AUC, Area under curve; CI, Confidence interval; CURB-65, Confusion, urea nitrogen, respiratory rate, blood pressure, and age ≥ 65 years; FDR, False discovery rate; FPR, False positive rate; HR, Hazard ratio; ICP, Intracranial pressure; IL-1RA, Interleukin 1 receptor antagonist; ROC, Receiver operating characteristic; TPR, True positive rate

Following univariate analyses encompassing clinical features and cytokines, we constructed multivariate prediction models utilizing COX regressions to anticipate potential factors associated with 3-year survival. The results, as demonstrated in Fig. 3A and Supplementary Fig. 4, revealed significant effects on survival time after diagnosis for independent factors such as dyspnea, ICP values ranging from 181 to 330mmH_2_O, severe GCS scores, and high-risk CURB-65 scores. Utilizing RCS analysis, we identified cutoff values of 3.5, 1.8, and 0.77 (after log_10_ transformations) at which the impact of IL-1RA, MCP-1, and IL-6 cytokines on 3-year survival changed (Fig. 3B). Through COX regression analysis, a total of four combined models including IL-1RA, IL-6, CURB-65 (high risk), and CURB-65 (low risk) were constructed stepwise (Fig. 3C). Among that, we verified a significant association between log_10_ IL-1RA (*p*-values: 0.01; hazard ratio: 2.1–77) and 3-year mortality, with similar findings observed in other combined models. ROC curve evaluation (Fig. 3C, D) indicated that multivariate models 1, 3, and 4 exhibited high accuracy (AUC = 0.95/0.96/0.96) in predicting 1-year mortality. Model 1 and model 3 emerged as cost-effective diagnostic potentials, maintaining a stable performance above 0.7 for fatalities of more than 2 years (Fig. 3D) when compared to other models. Consequently, the data suggest that the multivariate model involving IL-1RA (model 1) and the combination of IL-1RA, IL6, and high-risk CURB-65 has exceptional discriminative power in predicting both short- and long-term survival time.

### Longitudinal outcome assessments of ARTs and antifungal treatments

**Fig. 4:**
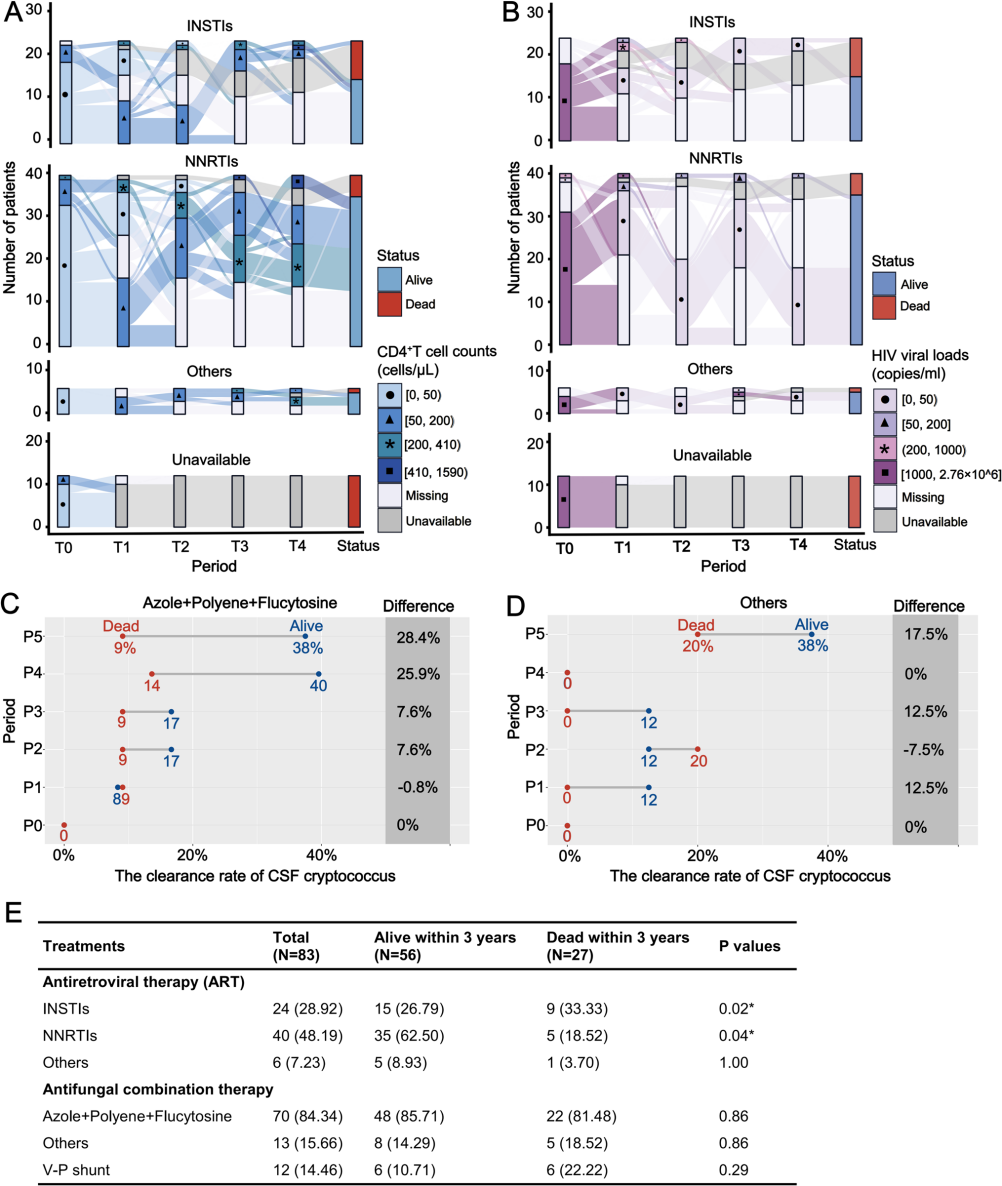
Longitudinal assessment of dynamic follow-up treatment efficacy. **a, b** Alluvium plot visualizing the distributions and dynamic comparisons for CD4^+^ T cell counts (**a**) and HIV viral loads (**b**) at baseline (T0) with different follow-up periods (T1, T2, T3, and T4) before or after different combinations of antiretroviral therapies (ARTs). INSTIs refer to two NRTIs with one INSTI, while NNRTIs represent two NRTIs and one NNRTI. Other ART regimens include two NRTIs with one PI, or combinations of NRTI and NNRTI with PI. T1 represents the first follow-up time point after the initial ART within 6 months; T2, T3, and T4 represent subsequent follow-ups within 12, 24, and 36 months, respectively. Unavailable indicates that the patient dead during the follow-up period, and the data cannot be obtained. **c, d** Contrasts for the clearance rate of CSF Cryptococcus at baseline (P0) and at different follow-ups (P1–P5) between 3-year survivors and non-survivors before or after different classes of antifungal therapies. Except for the classic antifungal therapy combination (azole + flucytosine + polyene) (**c**), all other antifungal therapy classes were grouped under “others” (**d**), which includes azole + flucytosine, azole + polyene, and polyene + flucytosine. The time points for follow-up assessments were defined as follows: P1 indicates the first follow-up assessment within 14 days after antifungal treatments; P2, P3, P4, and P5 indicate follow-up assessments within 1 month, 2 months, 6 months, and 12 months after treatment, respectively. **e** Comparisons for different ARTs and different antifungal treatments between the alive and dead over 3 years. *P*-values are calculated by Chi-square test (or Fisher’s exact test as appropriate) (*p < 0.05). Abbreviations: CSF, Cerebrospinal fluid; INSTIs, Integrase strand transfer inhibitors; MCP-1, Monocyte chemoattractant protein-1; NRTIs, Nucleoside reverse transcriptase inhibitors; NNRTIs, Non-nucleoside reverse transcriptase inhibitors; PIs, Protease inhibitors; V-P, ventriculoperitoneal

To evaluate the outcomes associated with both treatments, we conducted a comprehensive longitudinal analysis encompassing 3-year ART and 1-year antifungal treatments. This study aimed to provide insights into these interventions’ effectiveness and comparative efficacy over time. In the 1st year of treatment (Fig. 4A, B), when excluding missing data and deceased individuals, NNRTI-based regimens exhibited a higher rate of patients with increase in CD4^+^ T cell counts over 200 cells/μL compared to INSTI-based regimens (25.83% vs 9.09%). A similar trend was observed for achieving plasma HIV viral loads below 50 copies/ml (92.21% vs 87.50%). Supplementary Fig. 5 also reveals that, except for non-survivors, approximately 32.14% (9/28) of patients treated with NNRTIs regimens achieved CD4^+^ T cell counts exceeding 350 cells/μL within 3 years. In terms of antifungal treatments, the standard regimen consisting of azole, polyene, and flucytosine was administered to the majority of patients (84.34%, Fig. 4E). The CSF cryptococcal clearance rates in survivors consistently increased during treatment with azole, polyene, and flucytosine (Fig. 4C, D). However, there were unstable differences in clearance rates among the survival groups when other combined antifungal regimens were used at different follow-up points.

## Discussion

This study enrolled untreated HIV-associated disseminated cryptococcosis involving the lungs and CNS, utilizing multidimensional data and multiple-variate regression models to predict 3-year mortality risk and explore longitudinal treatment dynamics. Given the intricate nature of diagnosing and treating HIV-related disseminated cryptococcosis, the utilization of comprehensive and multiple-variate predictions proves more practical in serving as potential risk biomarkers for clinical prognostication, surpassing the conventional reliance on single clinical factors.

In our cohort, the 1-year mortality was 12.5%, and the 3-year mortality rate was 32.53%, both significantly lower than the previously reported 1-year mortality rate of 78% in a Malawian cohort [6]. The data demonstrate that mortality associated with HIV-related CM has been reduced with effective ART and antifungal therapy [22]. Results from this cohort reveal a male predominance, consistent with previous findings on the higher prevalence of HIV infection among males and their increased susceptibility to Cryptococcus [23, 24]. Independent risk factors for mortality in HIV-associated cryptococcal meningitis (HCM) include low CD4^+^ T cell counts, advanced age, and low body weight [22]. However, our results indicate no significant differences in CD4^+^ T cell counts, age, and body mass index (BMI) between survivors and non-survivors within 3 years.

Interleukin-1 (IL-1) is a pro-inflammatory cytokine crucially involved in infection-related inflammation [25]. Comprising two distinct cytokine peptides, IL-1α and IL-1β, IL-1 signaling is regulated by the IL-1 receptor antagonist (IL-1RA), which competitively binds to IL-1R1, exerting control over the inflammatory response [25]. During the early stages of HIV infection, it has been demonstrated that HIV can induce the production of IL-1RA [26]. Recent evidence demonstrates the ability of IL-1RA, IL-1α, and IL-1β to cross the blood–brain barrier through a saturable mechanism [27]. Preclinical studies have revealed the potential of IL-1RA drugs, such as anakinra, as a protective treatment for brain injury in cases of acute stroke and HIV-related disseminated tuberculosis [25, 28]. In the context of disseminated cryptococcosis with HIV, elevated IL-1RA levels in the blood and CSF maybe resulted from inefficient and localized attempts to inhibit IL-1 actions, thereby contributing to inflammation-induced damage and increased mortality rates. Further investigations are needed to validate this hypothesis. Notably, multivariate survival analysis also identifies IL-1RA as a stable potential short- and long-term mortality risk factor in this cohort. In light of the expectation, the elevation of IL-1RA as an unsuccessful attempt to reduce IL-1 levels, adjunct medical treatment involving IL-1RA may offer practical benefits for the coinfection of HIV and cryptococcosis affecting the lung and brain, which need to be demonstrated with more advanced research. IL-1 activates intracellular signaling, increasing the expression of the systemic acute-phase response cytokines, such as IL-6; however, the signaling can be blocked down by IL-1RA [25]. It may explain the instability of model 2 in predicting death.

MCP-1 (CCL2) increases the permeability of the blood–brain barrier and an augmented Cryptococcus burden in the CSF [29, 30]. CCR2, the receptor of CCL2, is present in neurons. MCP-1/CCR2 pathway is a critical pathway driving neuroinflammation, especially inflammatory monocyte recruitment, as well as CNS pathology and mortality in CM mice [31]. We observed a significant increase in plasma MCP-1 expression among the deceased group compared to the surviving group, while CSF MCP-1 demonstrated a similarly upward trend without statistical significance. Elevated baseline CNS expression of MCP-1 was associated with subsequent immune reconstitution inflammatory syndrome development in HIV-associated CM [32]. In addition, elevated MCP-1 also contributes to neurotropism and neurovirulence of HIV [33]. Inhibition of MCP-1 may provide a new therapy for HIV-associated disseminated cryptococcosis. IL-10 is predominantly secreted by Th2 cells during HIV infection, and a shift from Th1 toward Th2 response occurs, impairing the cellular immune response. Previous investigations have indicated a positive correlation between elevated levels of the anti-inflammatory cytokine IL-10 in CSF, the severity of disseminated infection, and a higher CSF fungal burden [34–36]. Our study observed an association between increased IL-10 levels and survival. In addition, elevated IL-10 levels have been associated with the progression of HIV [37]. The dysregulation of Th2 responses may be related to advancing HIV-associated disseminated cryptococcosis; further studies are required to validate these findings. Interestingly, several cytokines displayed opposing trends in plasma and CSF between the two groups. These findings suggest a lack of correlation between the release of cytokines and chemokines upon leukocyte activation in plasma and CSF in the context of HIV-associated disseminated cryptococcosis.

Notably, patients with ICP values ranging from 181 to 330 mmH_2_O exhibited a significantly shorter survival time than those with ICP values exceeding 330 mmH_2_O. No significant differences were found in the time elapsed from symptom onset to diagnosis and received immediate antifungal treatment between these two groups. Previous studies have demonstrated that sustained elevations in ICP within the range of 204–272 mmH_2_O are associated with unfavorable clinical outcomes [38]. Similarly, other studies have reported that less extreme ICP values can impede the efficient delivery of crucial nutrients to the brain, thereby compromising prognosis. However, the relevant ICP threshold remains to be determined [39]. Consequently, we postulate that the increased mortality and poorer survival observed in individuals with ICP values ranging from 181 to 330 mmH_2_O may be attributed to the prolonged time required for ICP reduction after timely treatment, leading to irreversible intracranial damage. Considering previous findings indicated that therapeutic lumbar puncture (LP) reduces overall mortality, regardless of baseline ICP values or symptoms [40–42]. It is conceivable that another conjecture about the frequency of therapeutic LPs is related to this outcome. It is plausible that patients with middle ICP values, despite similar severity, are less likely to receive aggressive therapeutic LPs compared to those in the highest ICP group, potentially contributing to increased mortality rates. Further investigation is warranted to explore this hypothesis. The CURB-65 score is a widely recognized clinical prediction tool used to assess mortality risk in hospitalized patients with community-acquired pneumonia [10]. The high-risk CURB-65 score group exhibited significantly shorter survival time compared to the medium-risk and low-risk groups. Previous studies have highlighted the predictive value of the CURB-65 score in assessing the 6-month mortality risk associated with HIV-related opportunistic infections, particularly pneumocystis pneumonia [43]. Our multivariate predictive model further showcased the enduring predictive capability of the CURB-65 score in evaluating both short-term and long-term mortality risks, in conjunction with other variables, in the context of HIV-disseminated cryptococcal disease.

Our observations indicated that the NNRTI-based regimen may show greater benefits than those of INSTIs in reducing HIV RNA levels, increasing CD4^+^ T cell counts, and reducing mortality. However, previous investigations have predominantly recommended using INSTI-based regimens as the primary therapeutic approach for HIV treatment [44]. We speculate that HIV-associated cryptococcosis may cause env and gag gene mutations, aggravate the multi-infection of HIV, and lead to INSTI resistance [45, 46]. Untreated high CSF fungal burden is a potent risk factor for mortality, with adjunctive antifungal therapy facilitating cryptococcal clearance. Combination antifungal therapy with azoles, polyenes, and flucytosine showed more stable CSF fungal clearance in our cohort.

There are several limitations to this study. First, the retrospective and insufficient sample size might have led to a failure to detect some crucial predictors. It is also hard to gain insights into the immune response profile in CNS owing to the lack of enough CSF sample size. Due to the exclusion of some patients with severely incomplete data, there might be an overestimated mortality rate. The absence of early fungicidal activity (EFA) data limits our ability to comprehensively evaluate the efficacy of the antifungal treatments. Second, we only collected data from a single assessment of neurological deficits. We did not follow up longitudinally on the dynamic changes of ICP levels and long-term CM-related neuro-sensorial impairment and disability. Finally, at a follow-up exceeding 2 years, the data about CD4^+^ T cell counts and plasma HIV viral load in the deceased group were missing due to patient death; however, our results should be valuable for future prospective studies.

## Conclusions

Significantly elevated plasma IL-1RA and MCP-1 levels were observed in non-survivors compared to survivors. The present study emphasizes the importance of IL-1RA as a potential biomarker linked to the risk of mortality over a 3-year timeframe. Further investigations to explore the pathogenetic role of IL-1RA in HIV-associated disseminated cryptococcosis and as a potential therapeutic target are warranted.

## Supplementary Information

Below is the link to the electronic supplementary material.

Supplementary file1 (DOCX 30 KB)

Supplementary file2 (DOCX 650 KB)

Supplementary file3 (DOCX 14 KB)

## Data Availability

No datasets were generated or analysed during the current study.
