# Correction: Comprehensive risk factor predictions for 3-year survival among HIV-associated and disseminated cryptococcosis involving lungs and central nervous system

**DOI:** 10.1007/s15010-024-02281-2

**Published:** 2024-05-17

**Authors:** Luling Wu, Xuemin Fu, Benno Pütz, Renfang Zhang, Li Liu, Wei Song, Ling Weng, Yueming Shao, Zhihang Zheng, Jingna Xun, Ximei Han, Ting Wang, Yinzhong Shen, Hongzhou Lu, Bertram Müller-Myhsok, Jun Chen

**Affiliations:** 1 Institute of Antibiotics, Huashan Hospital, Fudan University, Shanghai, China; 2 Department of Infectious Diseases and Immunology, Shanghai Public Health Clinical Center, Fudan University, Shanghai, China; 3 Research Group Statistical Genetics, Max Planck Institute of Psychiatry, Munich, Germany; 4 Department of Respiratory Medicine, Fuzhou Pulmonary Hospital, Fuzhou, Fujian China; 5 Department of Infectious Diseases and Nursing Research Institution, National Clinical Research Center for Infectious Diseases, The Third People’s Hospital of Shenzhen, Shenzhen, China

## **Correction: Infection**10.1007/s15010-024-02237-6

Unfortunately, there are some mistakes with the affiliations of the following authors, including Renfang Zhang, Li Liu, Wei Song, Yueming Shao, Zhihang Zheng, Jingna Xun, Yinzhong Shen, and Jun Chen, which should be affiliated with the “2. Department of Infectious Diseases and Immunology,Shanghai Public Health Clinical Center, Fudan University, Shanghai, China”. We are very sorry for causing this misunderstanding and inconvenience. All authors contributed to the work, participated in the review process, and have given their approvals for submission.

Luling Wu^1,2^ · Xuemin Fu^3^ · Benno Pütz^3^ · Renfang Zhang^1^ · Li Liu^1^ · Wei Song^1^ · Ling Weng^4^ · Yueming Shao^1^ · Zhihang Zheng^1^ · Jingna Xun^1^ · Ximei Han^4^ · Ting Wang^4^ · Yinzhong Shen^1^ · Hongzhou Lu^5^ · Bertram Müller Myhsok^3^ · Jun Chen^1^

^1^Institute of Antibiotics, Huashan Hospital, Fudan University, Shanghai, China

^2^Department of Infectious Diseases and Immunology, Shanghai Public Health Clinical Center, Fudan University, Shanghai, China

^3^Research Group Statistical Genetics, Max Planck Institute of Psychiatry, Munich, Germany

^4^Department of Respiratory Medicine, Fuzhou Pulmonary Hospital, Fuzhou, Fujian, China

^5^Department of Infectious Diseases and Nursing Research Institution, National Clinical Research Center for Infectious Diseases, The Third People’s Hospital of Shenzhen, Shenzhen, China

Correct is:

Luling Wu^1,2†^, Xuemin Fu^3†^, Benno Pütz^3^, Renfang Zhang^2^, Li Liu^2^, Wei Song^2^, Ling Weng^4^, Yueming Shao^2^, Zhihang Zheng^2^, Jingna Xun^2^, Ximei Han^4^, Ting Wang^4^, Yinzhong Shen^2^, Hongzhou Lu^5^, Bertram Müller-Myhsok^3*^, Jun Chen^2*^

^1^Institute of Antibiotics, Huashan Hospital, Fudan University, Shanghai, China

^2^Department of Infectious Diseases and Immunology, Shanghai Public Health Clinical Center, Fudan University, Shanghai, China

^3^Research Group Statistical Genetics, Max Planck Institute of Psychiatry, Munich, Germany

^4^Department of Respiratory Medicine, Fuzhou Pulmonary Hospital, Fuzhou, Fujian, China

^5^Department of Infectious Diseases and Nursing Research Institution, National Clinical Research Center for Infectious Diseases, The Third People's Hospital of Shenzhen, Shenzhen, China

The Original Article has been corrected.

