# Supplementary material for: Comprehensive risk factor predictions for 3-year survival among HIV-associated and disseminated cryptococcosis involving lungs and central nervous system
Source: Infection. 2024 Apr 13;52(5):1875–87. doi: 10.1007/s15010-024-02237-6 (PMC11499439; doi:10.1007/s15010-024-02237-6)
Supplement: Supplementary file 1 — Supplementary file1 (DOCX 30 KB) [file 15010_2024_2237_MOESM1_ESM.docx]

|  | **Total (N=83)** | **Alive within 3 years (N=56)** | **Dead within 3 years (N=27)** | **P values** |
| --- | --- | --- | --- | --- |
| **Clinical characteristics** | | | | |
| **Constitutional symptoms** | |  |  |  |
| Fever | 58 (69.88) | 40 (71.43) | 18 (66.67) | 0.66 |
| Vomiting | 27 (32.53) | 20 (35.71) | 7 (25.93) | 0.37 |
| Nausea | 24 (28.92) | 19 (33.93) | 5 (18.52) | 0.15 |
| Weight loss | 17 (20.48) | 10 (17.86) | 7 (25.93) | 0.39 |
| Weakness | 14 (16.87) | 9 (16.07) | 5 (18.52) | 1.00 |
| Anorexia | 7 (8.43) | 5 (8.93) | 2 (7.41) | 1.00 |
| Shiver | 5 (6.02) | 4 (7.14) | 1 (3.70) | 1.00 |
| **Respiratory symptoms** | |  |  |  |
| Sputum | 13 (15.67) | 6 (10.71) | 7 (25.93) | 0.14 |
| Cough | 6 (7.23) | 4 (7.14) | 2 (7.41) | 1.00 |
| Dyspnea | 6 (7.23) | 2 (3.57) | 4 (14.81) | 0.08 |
| Chest distress | 3 (3.61) | 1 (1.79) | 2 (7.41) | 0.25 |
| Hemoptysis | 3 (3.61) | 1 (1.79) | 2 (7.41) | 0.25 |
| Moist rales | 3 (3.61) | 1 (1.79) | 2 (7.41) | 0.25 |
| Chest pain | 1 (1.20) | 0 (0) | 1 (3.70) | 0.33 |
| Dry rales | 1 (1.20) | 0 (0) | 1 (3.70) | 0.33 |
| **Neurological symptoms** | |  |  |  |
| Headache | 38 (45.78) | 26 (46.43) | 12 (44.44) | 0.87 |
| Neck resistance | 21 (25.30) | 15 (26.79) | 6 (22.22) | 0.65 |
| Dizziness | 17 (20.48) | 13 (23.21) | 4 (14.81) | 0.37 |
| Conscious disturbance | 16 (19.28) | 12 (21.43) | 4 (14.81) | 0.47 |
| Hyperspasmia | 14 (16.87) | 12 (21.43) | 2 (7.41) | 0.12 |
| Vision disorders | 10 (12.05) | 6 (10.71) | 4 (14.81) | 0.86 |
| Signs of meningeal irritation | 10 (12.05) | 6 (10.71) | 4 (14.81) | 0.86 |
| Hemiplegia | 8 (9.64) | 3 (5.36) | 5 (18.52) | 0.13 |
| Hearing loss | 7 (8.43) | 5 (8.93) | 2 (7.41) | 1.00 |
| Muscle weakness | 2 (2.41) | 1 (1.79) | 1 (3.70) | 0.55 |
| **Asymptomatic** | 29 (34.94) | 19 (33.93) | 10 (37.04) | 0.78 |
| **HIV-associated opportunistic infection** | | | | |
| Talaromyces marneffei | 1 (1.20) | 0 (0) | 1 (3.70) | 0.33 |
| **Sites of involvement** | |  |  | 0.65 |
| Lung+CNS+Blood^b^ | 49 (59.04) | 34 (60.71) | 15 (55.56) |  |
| Lung+CNS^c^ | 34 (40.96) | 22 (39.29) | 12 (44.44) |  |
| **Exposure history**  **(pigeon droppings)** | 1 (1.20) | 1 (1.79) | 0 (0) | 1.00 |
| **PCT, ng/mL, median**  **(IQR) (N=75/49/26)^a^** | 0.06 (0.04-0.18) | 0.06 (0.04-0.16) | 0.06 (0.04-0.21) | 0.85 |
| **TBil, μmol/L, mean (SD)** | 10.59 (4.57) | 10.27 (4.16) | 11.26 (5.34) | 0.58 |
| **AST, U/L, mean (SD)** | 31.59 (24.09) | 33.52 (27.02) | 27.59 (16.20) | 0.62 |
| **ALT, U/L, mean (SD)** | 28.16 (16.12) | 28.18 (15.15) | 28.11 (18.28) | 0.75 |
| **Cr, μmol/L, mean (SD)** | 59.64 (25.55) | 61.25 (28.21) | 56.31 (18.93) | 0.24 |
| **BUN, mmol/L,**  **median (IQR)** | 4.10 (3.39-5.81) | 4.44 (3.41-5.78) | 3.90 (3.10-6.30) | 0.40 |
| **LDH , U/L, mean (SD)** | 260.39 (124.55) | 251.38 (105.75) | 279.07 (157.25) | 0.49 |
| **Serum Glucose, mmol/L, mean (SD)** | 6.59 (2.04) | 6.59 (2.15) | 6.59 (1.80) | 0.66 |
| **CSF white blood cell, g/L, median (IQR) (N=81/56/25)^a^** | 5 (2.50-12) | 6 (4-12) | 5 (2-17.50) | 0.58 |
| **CSF red blood cell, g/L, median (IQR) (N=81/56/25)^a^** | 0 (0- 2.75) | 0 (0-3) | 0 (0-2) | 0.99 |

**Supplementary Table 1. Comparisons of clinical features in untreated HIV-associated with disseminated cryptococcosis patients with lungs and CNS involvement between survivors and non-survivors.**

a. Available number of patients: total /survived 3 years /dead within 3 years.

b. Lung+CNS+Blood indicates the positive cryptococcal culture or positive CrAg titers in the lungs and CNS, besides a positive blood culture.

c. Lung+CNS represents that a positive cryptococcal culture or positive CrAg titers were detected in the lungs and CNS, without the positive blood culture.

d. Categorical variables are presented as number (frequency %).

e. *P*-values are calculated by Mann-Whitney U for continuous variables and Chi-square test (or Fisher’s exact test as appropriate) for categorical variables.

Abbreviations: ALT, Alanine Aminotransferase; AST, Aspartate Transaminase; BUN, Blood Urea Nitrogen; CNS, Central Nervous System; Cr, Creatinine; CSF, Cerebrospinal Fluid; LDH, Lactate Dehydrogenase; PCT, Procalcitonin; TBil, Total Bilirubin.

| **Variables (N_T1_)** | **Hazard Ratio (95% CI)** | **P values** | **FDR** |
| --- | --- | --- | --- |
| Log_10_ IL-1RA | 13 (2.10-77) | 0.01* | 0.022 |
| Log_10_ IL-6 | 12 (1.80-84) | 0.01* | 0.022 |
| CURB-65 score (High risk) | 4.50 (1.20-17) | 0.03* | 0.030 |
| CURB-65 score (Low risk) | 0.30 (0.08-0.86) | 0.03* | 0.030 |
| Log_10_ IL-8 | 22 (1.10-410) | 0.04* | 0.032 |
| Log_10_ MCP-1 | 4.80 (1-22) | 0.04* | 0.033 |
| ICP values (180-330mmH_2_O) | 2.30 (0.60-9) | 0.22 | 0.107 |

**Supplementary Table 2. Univariate analysis of clinical characteristics, cytokine and chemokine levels with significant differences.**

The *p* values are evaluated with the log-rank test. The FDR values are adjusted for *p*-values. N_T1_ means a training set (N_T1_=36) (* *p*<0.05).

Abbreviations: CURB-65, Confusion, Urea nitrogen, Respiratory rate, Blood pressure and Age≥65 years; FDR, False Discovery Rate; MCP-1, Monocyte Chemoattractant Protein-1; ICP, Intracranial Pressure; IL-1RA, Interleukin 1 Receptor Antagonist.
