# Supplementary material for: Comprehensive risk factor predictions for 3-year survival among HIV-associated and disseminated cryptococcosis involving lungs and central nervous system
Source: Infection. 2024 Apr 13;52(5):1875–87. doi: 10.1007/s15010-024-02237-6 (PMC11499439; doi:10.1007/s15010-024-02237-6)
Supplement: Supplementary file 2 — Supplementary file2 (DOCX 650 KB) [file 15010_2024_2237_MOESM2_ESM.docx]

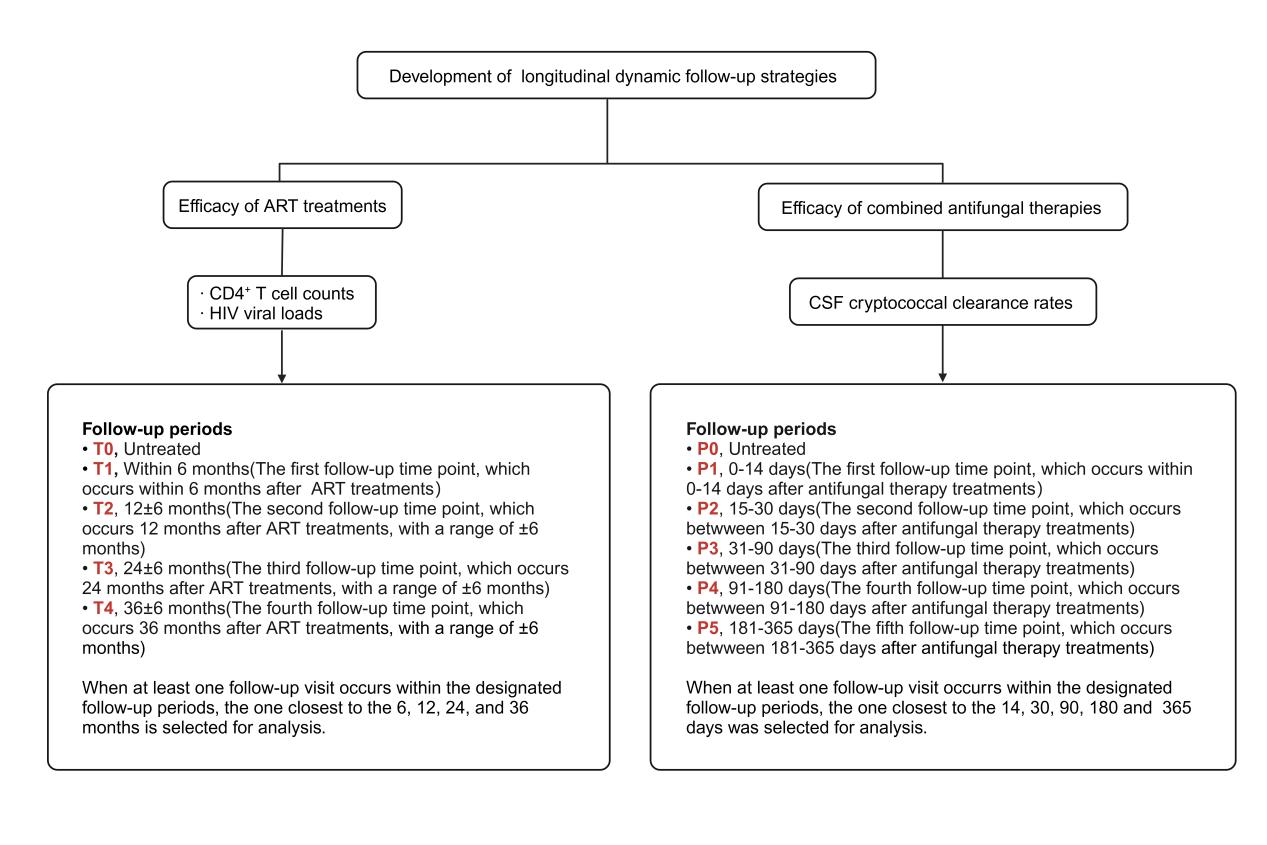


**Supplementary Fig. 1. Workflow diagram illustrates the process of implementing comprehensive longitudinal follow-up strategies.**

Abbreviations:ART, Antiretroviral Therapy; CSF, Cerebrospinal Fluid.


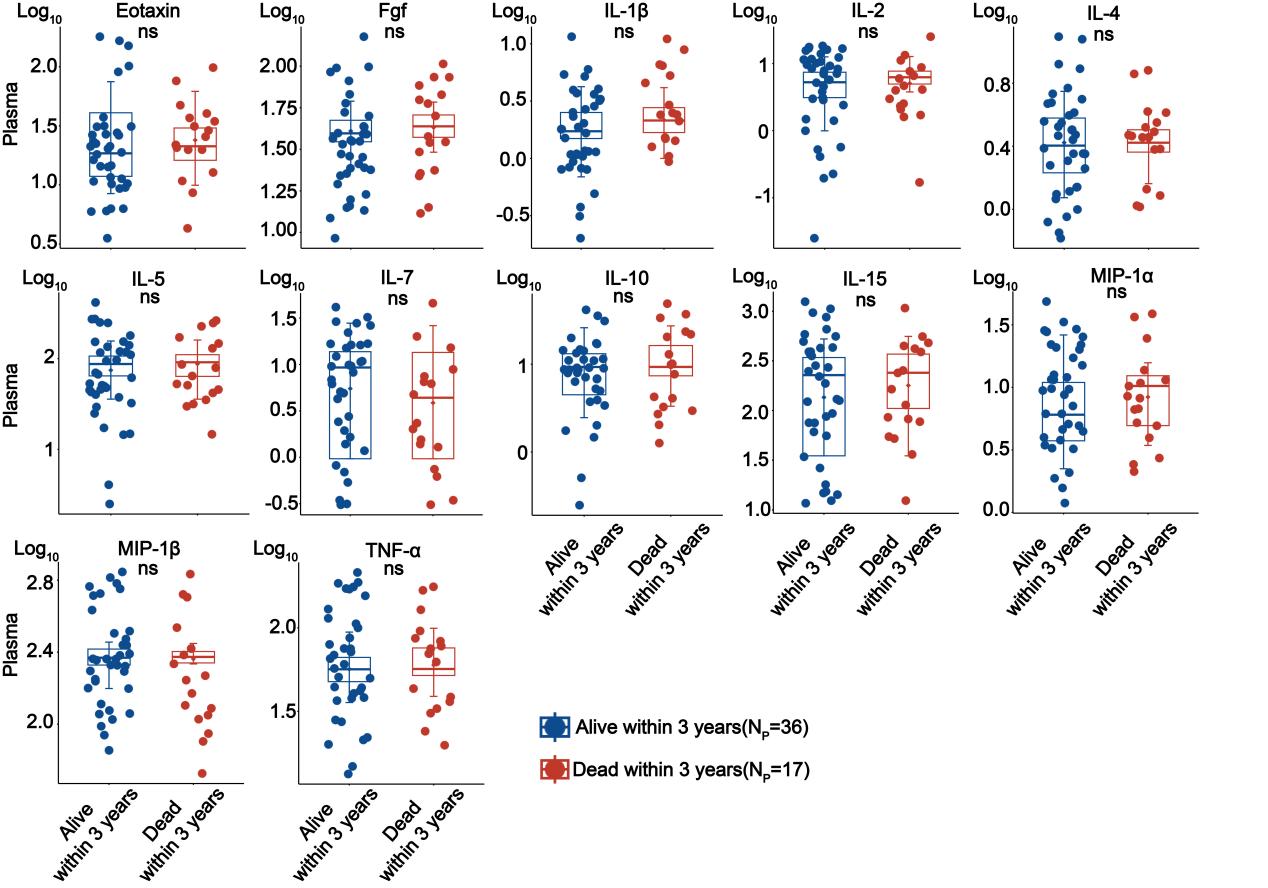


**Supplementary Fig. 2. Comparisons of plasma cytokines and chemokines before treatment between patients in the 3-year surviving and deceased patients.**

The cytokine and chemokine levels are converted to log10 values and compared in units of per milliliter (pg/ml), and *p*-values are calculated using the T-test (ns means not significant).

Abbreviations: Fgf, Fibroblast Growth Factor; MIP-1α, Macrophage Inflammatory Protein 1 Alpha; MIP-1β, Macrophage Inflammatory Protein 1 Beta; TNF-α, Tumor Necrosis Factor Alpha.


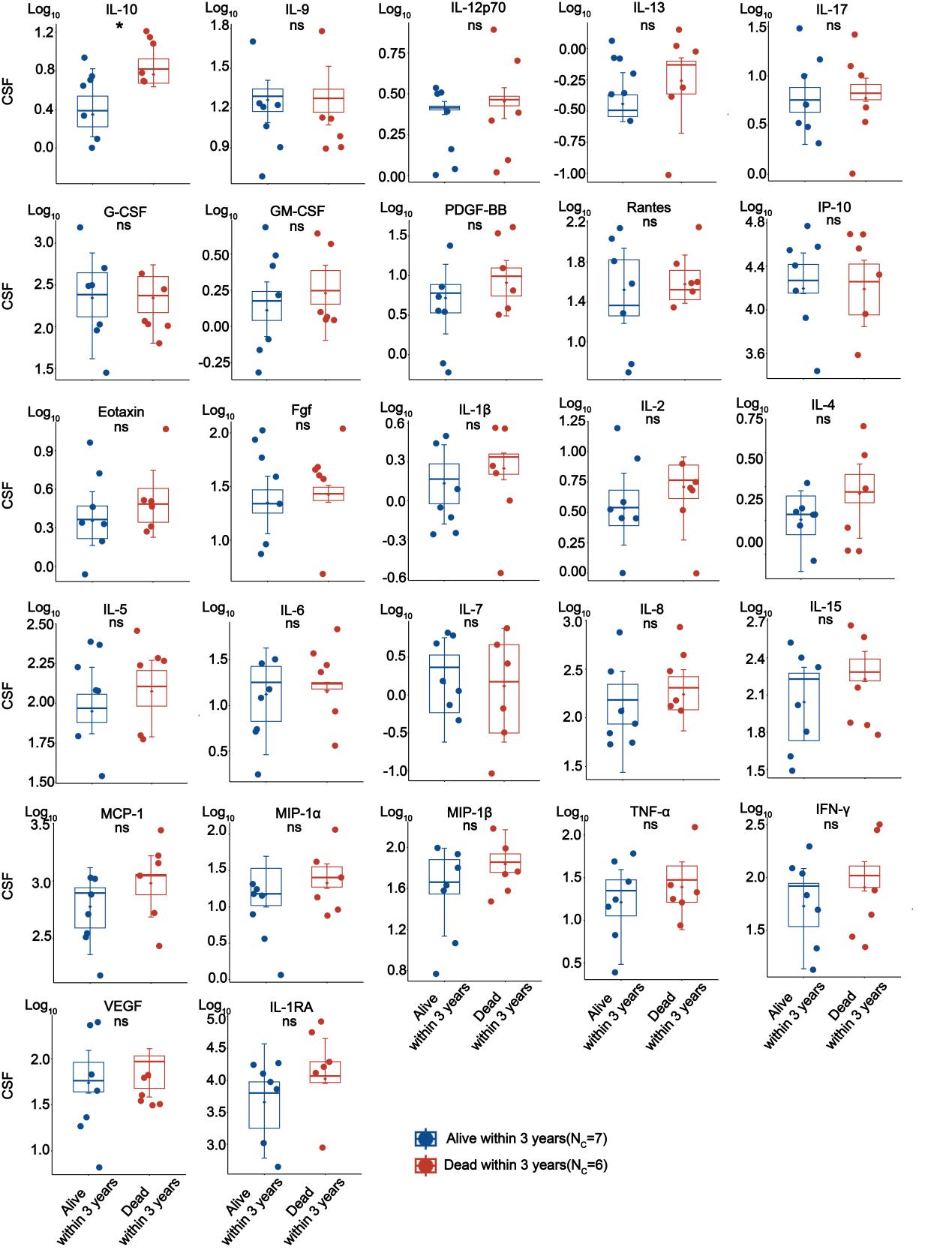


**Supplementary Fig. 3. Comparisons of cytokine and chemokine levels in CSF before treatment between the alive and dead groups within 3 years.**

The cytokine and chemokine levels are transformed logarithmically before comparison with picograms per milliliter (pg/ml), and all *p* values are calculated with the T test (* *p*<0.05; ** *p*<0.01; *** *p*<0.001, ns means not significant).

Abbreviations: CSF, cerebrospinal fluid; Fgf, Fibroblast Growth Factor; G-CSF, Granulocyte Colony-stimulating Factor; GM-CSF, Granulocyte-macrophage Colony Stimulating Factor; IL-1RA, Interleukin 1 Receptor Antagonist; IP-10, Interferon-inducible Protein-10; MCP-1, Monocyte Chemoattractant Protein-1; MIP-1α, Macrophage Inflammatory Protein 1 Alpha; MIP-1β, Macrophage Inflammatory Protein 1 Beta; PDGF-BB,  Platelet-derived Growth Factor-BB; TNF-α, Tumor Necrosis Factor Alpha; VEGF, Vasoactive Endothelial Growth Factor.


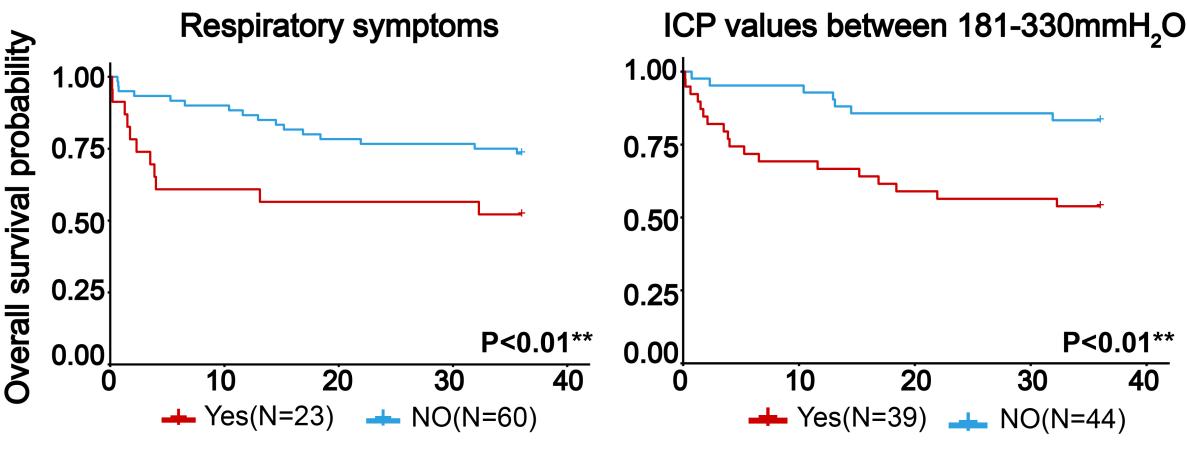


**Supplementary Fig. 4. Survival analysis with the Kaplan-Meier (KM) estimator based on clinical characteristics with significant differences in univariate analysis.**

*P*-values are determined using the log-rank test (** *p*<0.01).

Abbreviations: ICP, Intracranial Pressure.


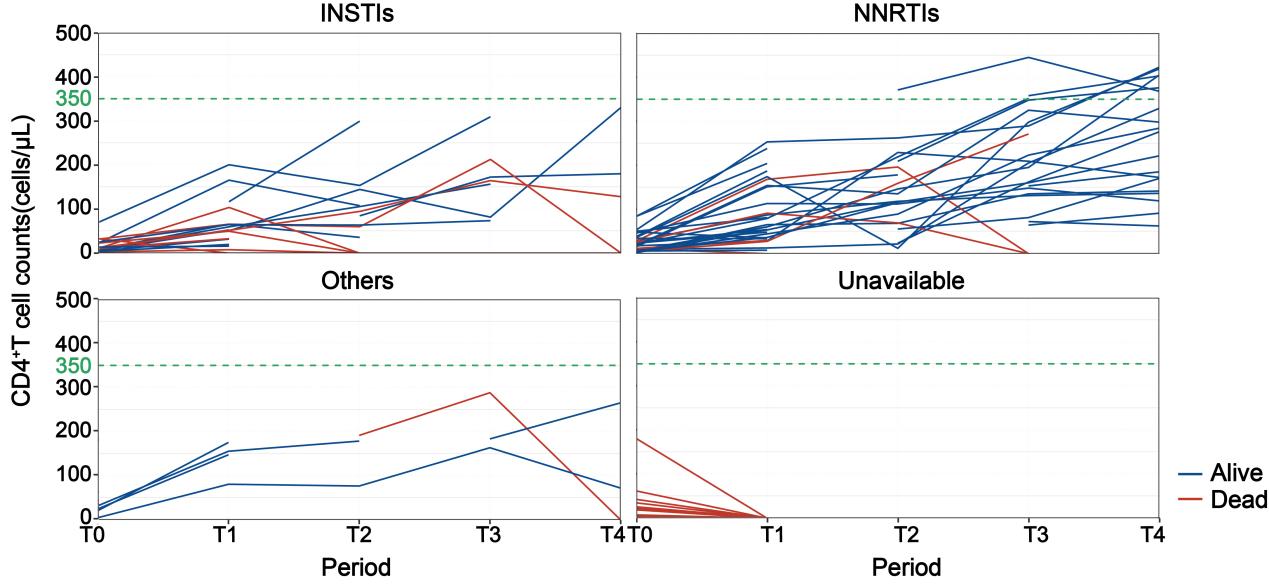


**Supplementary Fig. 5: Line graph showing CD4^+^ T cell counts before and after different ART treatments at different time periods.**

If CD4^+^ T cell counts could not be obtained due to patient death before the T4 period, the corresponding Y-axis value is below zero. The INSTIs regimen consists of a combination of two NRTIs and one INSTI, while NNRTIs refer to a regimen of two NRTIs and one NNRTI. Other types of ART include a combination of two NRTIs with one PI, or a combination of NRTI and NNRTI with PI. T0 refers to the time before ARTs. T1, T2, T3, and T4 correspond to the first follow-up time point within six months after ARTs, and subsequent follow-up time points at 12 months, 24 months, and 36 months, respectively. Unavailable indicates that data were not available due to patient death during the follow-up periods.

Abbreviations: INSTIs, Integrase Strand Transfer Inhibitors; NRTIs, Nucleoside Reverse Transcriptase Inhibitors; NNRTIs, Nonnucleoside Reverse Transcriptase Inhibitors; PIs, Protease Inhibitors.
