# Supplementary material for: Comprehensive risk factor predictions for 3-year survival among HIV-associated and disseminated cryptococcosis involving lungs and central nervous system
Source: Infection. 2024 Apr 13;52(5):1875–87. doi: 10.1007/s15010-024-02237-6 (PMC11499439; doi:10.1007/s15010-024-02237-6)
Supplement: Supplementary file 3 — Supplementary file3 (DOCX 14 KB) [file 15010_2024_2237_MOESM3_ESM.docx]

**SUPPLEMENTARY METHOD**

**Antifungal therapy strategy**The antifungal therapy regimen follows the World Health Organization guidelines for cryptococcal treatment[1]. All antifungal drugs were initiated as soon as the participants were diagnosed with cryptococcosis, combined with active intracranial pressure-lowering treatment. Patients received at least 4 weeks of amphotericin B (AmB) and 5-fluorocytosine during the induction phase. In patients who were intolerant to AmB, voriconazole or high doses of fluconazole were administrated. When the patient's clinical symptoms improved and the cerebrospinal fluid culture (CSF) turned negative, the treatment was changed to fluconazole for consolidation therapy. They are required to receive fluconazole treatment for more than 1 year until their CD4^+^ T cell counts exceed 100 cells/*μ*L with the initiation of antiretroviral therapy (ART). Mannitol was used in patients with high intracranial pressure (ICP) per recommendation of the Chinese guideline for cryptococcal treatment. When the patients' intracranial pressure continuously increases to or over 250 mm H_2_O, accompanied by the development or recurrence of symptoms of elevated intracranial pressure, they received repeat therapeutic lumbar puncture every other day or as the clinical symptoms indicated high ICP. Potassium was administrated intravenously or orally and monitored closely in patients receiving AmB.

**References**

1. World Health Organization. 2022. Guidelines for diagnosing, preventing and managing cryptococcal disease among adults, adolescents and children living with HIV. World Health Organization. Geneva. (2022). <https://apps.who.int/iris/handle/10665/357088>. Accessed.
